# Changes in Capsule and Drug Resistance of Pneumococci after Introduction of PCV7, Japan, 2010–2013

**DOI:** 10.3201/eid2007.131485

**Published:** 2014-07

**Authors:** Naoko Chiba, Miyuki Morozumi, Michi Shouji, Takeaki Wajima, Satoshi Iwata, Kimiko Ubukata, the Invasive Pneumococcal Diseases Surveillance Study Group

**Affiliations:** Keio University School of Medicine, Tokyo, Japan (N. Chiba, M. Morozumi, S. Iwata, K. Ubukata);; Kitasato University, Tokyo (N. Chiba, M. Morozumi, K. Ubukata);; National Cancer Center Hospital, Tokyo (M. Shouji);; Tokyo University of Pharmacy and Life Sciences, Tokyo (T. Wajima)

**Keywords:** 7-valent pneumococcal conjugate vaccine, 13-valent pneumococcal conjugate vaccine, PCV7, PCV13, capsular type, β-lactam resistance, macrolide resistance, invasive pneumococcal diseases, Japan, bacteria, Streptococcus pneumoniae

## Abstract

We aimed to clarify changes in serotypes and genotypes mediating β-lactam and macrolide resistance in *Streptococcus pneumoniae* isolates from Japanese children who had invasive pneumococcal disease (IPD) after the 7-valent pneumococcal conjugate vaccine (PCV7) was introduced into Japan; 341 participating general hospitals conducted IPD surveillance during April 2010–March 2013. A total of 300 pneumococcal isolates were collected in 2010, 146 in 2011, and 156 in 2012. The proportion of vaccine serotypes in infectious isolates decreased from 73.3% to 54.8% to 14.7% during the 3 years. Among vaccine serotype strains, genotypic penicillin-resistant *S. pneumoniae* strains also declined each year. Among nonvaccine serotype strains, 19A, 15A, 15B, 15C, and 24 increased in 2012. Increases were noted especially in genotypic penicillin-resistant *S. pneumoniae* isolates of serotypes 15A and 35B, as well as macrolide resistance mediated by the *erm*(B) gene in 15A, 15B, 15C, and 24.

## Abstract 2

Drastic changes threaten overall effectiveness of this vaccine.

Invasive pneumococcal disease (IPD), such as meningitis, sepsis, and empyema, substantially contributes to illness and death in children (*1*,*2*). After increasing numbers of cases caused by penicillin (PEN)–resistant *Streptococcus pneumoniae* (PRSP) emerged and rapidly spread worldwide during the 1990s (*3*,*4*), the need for a vaccine effective in infants became clear. In the United States, a 7-valent pneumococcal conjugate vaccine (PCV7) was introduced in 2000 and made available for routine use in all children 2–23 months of age and in children 24–59 months of age at risk for pneumococcal infection (*5*). Subsequent surveillance studies demonstrated a marked decrease in prevalence of pneumococcal infection caused by vaccine serotypes, including PRSP (*6*–*8*). In particular, PCV7 appears to have decreased incidence of meningitis caused by vaccine serotypes (*9*), and cases caused by non-PCV7 serotype strains, such as PRSP with serotype 19A, have increased in the United States (*8*,*10*,*11*). Such changes suggest that nonvaccine serotypes are replacing vaccine serotypes in some countries (*12*–*14*).

A next-generation 13-valent pneumococcal conjugate vaccine (PCV13) was licensed for use in the United States in 2010 (*15*). PCV13 has been approved in 128 countries, and children in 83 countries have undergone routine PCV13 vaccination. Recently, Richter et al. reported that an increase of type 19A was halted by introduction of PCV13, whereas serotype 35B increased; coverage provided by PCV13 was effective in only 41.4% of children <5 years of age in 2010 and 2011 (*16*). Furthermore, Kaplan et al. reported a slight increase in serotype 33F (*17*).

In Japan, PRSP has increased rapidly as a cause of respiratory tract infections, acute otitis media, and IPD in children since the late 1990s (*18*,*19*). PCV7 received final approval in October 2009 and has been used clinically in infants on a voluntary basis since February 2010. Since November 2010, PCV7 use has been encouraged for children <5 years of age throughout Japan by an official program, the Provisional Special Fund for the Urgent Promotion of Vaccination. As a result, estimated rates of PCV7 vaccination for such children were <10% in 2010, 50%–60% in 2011, and 80%–90% in 2012.

PCV7 was incorporated into the routine vaccination schedule for children in Japan beginning in April 2013; before then, however, its coverage rate against IPD had decreased rapidly from 71.8% in 2006 to 51.6% in 2011 (*20*). Most recently, PCV13 was approved by the government in June 2013, later replacing PCV7 as a routine vaccination in November 2013. The purpose of our study was to clarify changes during April 2010–March 2013 of serotypes and genotypes mediating β-lactam and macrolide resistance in *S. pneumoniae* isolates from children <18 years of age who had IPD before and after PCV7 introduction.

## Methods

### Patients and Pneumococcal Strains

All study participants were children <18 years of age who had IPD. Pneumococcal isolates from normally sterile clinical samples, such as blood, cerebrospinal fluid, pleural effusions, and joint fluid, were collected and examined.

**Figure 1:**
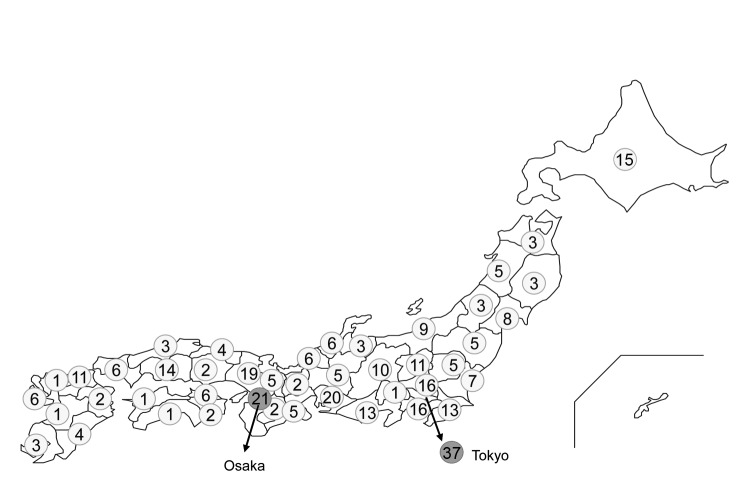
Distribution of general hospitals participating in surveillance for invasive pneumococcal disease, Japan, April 2010–March 2013. Numbers in light gray circles show the number of the hospitals in each prefecture. Numbers in dark gray circles show the number of hospitals in Tokyo and Osaka.

Medical institutions that had a microbiology laboratory and a pediatric department with hospitalization facilities were permitted to participate actively in surveillance. An estimated 25% of all general hospitals in Japan participated. Participating hospitals were distributed nearly uniformly throughout Japan (Figure 1). These hospitals took part in the surveillance project after the laboratory director or hospital director granted permission in writing. A questionnaire collecting information for every case-patient was completed anonymously in accordance with the ethical guidelines for conducting epidemiologic studies in Japan.

Pneumococcal isolates were collected nationwide during 3 periods. The first surveillance period was April 2010–March 2011, when voluntary vaccination with PCV7 was estimated to be <10% (vol-PCV7: 2010). The second period was April 2011–March 2012, when the estimated vaccination rate was 50%–60% because of the Urgent Promotion of Vaccination incentive (post-PCV7: 2011). The third period was April 2012–March 2013, when the vaccination rate was 80%–90% just before introduction of PCV13 (pre-PCV13: 2012). We collected a total of 602 isolates from 341 general hospitals: 300 in 2010, 146 in 2011, and 156 in 2012.

### Serotypes and Antimicrobial Drug–Resistant Genotypes

Serotypes of all isolates were determined by the capsular quellung reaction using antiserum purchased from the Statens Serum Institute (Copenhagen, Denmark). Alterations in 3 penicillin-binding protein (PBP) genes mediating β-lactam resistance in *S. pneumoniae*—*pbp1a* (PBP1A), *pbp2x* (PBP2X), and *pbp2b* (PBP2B)—were identified by real-time PCR methods that we developed and reported previously (*21*). The PCR system detected the presence of amino acid substitution(s) in conserved amino acid motif(s), such as serine-threonine-methionine-lysine, in each PBP. The genes *mef* (A) and *erm* (B), which confer resistance to macrolide antimicrobial drugs, also were identified by real-time PCR (*21*).

Genotype (g) based on molecular analysis is represented here as penicillin-susceptible *S. pneumoniae* (gPSSP) possessing 3 normal *pbp* genes; penicillin-intermediate *S. pneumoniae* (gPISP), further classified as gPISP (*pbp2x*), gPISP (*pbp1a+pbp2x*), or gPISP (*pbp2x+pbp2b*); or penicillin-resistant *S. pneumoniae* (gPRSP), possessing all 3 abnormal *pbp* genes (*22*). Relationships between susceptibilities to β-lactam agents among phenotypes of *S. pneumoniae* and resistance genotype were described previously (*21*). Genotypes involving macrolide resistance are represented here as the following: macrolide-susceptible *S. pneumoniae* not possessing any genes (MLS); intermediate macrolide resistance to 14- or 15-membered macrolides mediated by the *mef*(A) gene (MLR-*mef*[A]); high macrolide resistance to all macrolides mediated by the *erm*(B) gene (MLR-*erm*[B]); and high macrolide resistance possessing both genes (MLR-*mef*[A]+*erm*[B]).

For each strain transferred to our laboratory, we promptly identified capsular type by quellung reaction, and resistance genotype was determined by real-time PCR. Results were returned immediately to medical staff at the referring hospital. Medical personnel caring for the patients considered this surveillance and results reporting system very helpful, and it was continued for 3 years.

### Multilocus Sequence Typing

Multilocus sequence typing for the strains collected was performed according to previously described methods with slight modifications (*23*). Primers listed on the website of the Centers for Disease Control and Prevention (http://www.cdc.gov/ncidod/biotech/strep/alt-MLST-primers.htm) were used. Multilocus sequence typing and eBURST analyses were performed according to published methods (http://spneumoniae.mlst.net).

### Statistical Analysis

We assessed statistical significance of differences in serotype distribution between the 3 periods, age groups, and specific infectious diseases. We used χ^2^ tests or the Fisher exact test, using Ekuseru-Toukei 2012 software for statistics (Social Survey Research Information Co., Ltd., Tokyo, Japan).

## Results

### Patient Age and Coverage Rate by PCV7

Estimated rates of vaccination with PCV7 were <10% in 2010 (voluntary-PCV7) but rose to 50%–60% with funding in 2011 (post-PCV7) and to 80%–90% with enhanced implementation of PCV7 just before the transition from PCV7 to PCV13 in 2012 (pre-PCV13). *S. pneumoniae* isolates from patients with IPD, collected every year, decreased by half in 2011 and 2012 from those in 2010. In particular, vaccine serotypes decreased significantly among patients <4 years of age (p<0.001) but not among those >5 years of age (p = 0.733) (Table 1). For all patients, coverage rate by PCV7 decreased rapidly from 73.3% in 2010 to 54.8% in 2011 to 14.7% in 2012, and prevalence proportion of nonvaccine serotypes increased for 3 years (p<0.001).

**Table 1:** Vaccine and nonvaccine serotypes of *Streptococcus pneumoni*ae in children after introduction of PCV7, Japan, April 2010–March 2013*

Age group, y, and serotype	Isolates, no. (%)			p value
	2010, n = 300†	2011, n = 146‡	2012, n = 156§	
<1				<0.001
VT	51 (17.0)‡	26 (17.8)	3 (1.9)	
NVT	19 (6.3)	14 (9.6)	25 (16.0)	
1				<0.001
VT	103 (34.3)	25 (17.1)	9 (5.8)	
NVT	31 (10.3)	28 (19.2)	62 (39.7)	
2–4				<0.001
VT	55 (18.3)	23 (15.8)	6 (3.8)	
NVT	19 (6.3)	14 (9.6)	39 (25.0)	
>5				0.733
VT	11 (3.7)	6 (4.1)	5 (3.2)	
NVT	11 (3.7)	10 (6.8)	7 (4.5)	
Total				<0.001
VT	220 (73.3)	80 (54.8)	23 (14.7)	
NVT	80 (26.7)	66 (45.2)	133 (85.3)	

*PCV7, 7-valent pneumococcal conjugate vaccine; VT, vaccine serotype (serotypes 4, 6B, 9V, 14, 18C, 19F, 23F); NVT, serotypes not included in PCV7. †Voluntary vaccination with PCV7. ‡Implementation of Urgent Promotion of Vaccination incentive. §Just before the introduction of 13-valent PCV.

### Year-to-Year Changes in Vaccine and Nonvaccine Serotype Prevalence Proportion by Disease

We compared year-to-year changes in prevalence of vaccine and nonvaccine serotypes of S. pneumoniae in patients who had meningitis; sepsis and bacteremia; pneumonia; and other invasive infections, including cellulitis, arthritis, endocarditis, and empyema during each of the 3 years studied (Table 2). Pneumonia cases were included only when *S. pneumoniae* was isolated from blood cultures. Vaccine serotype strains decreased significantly in patients with meningitis (p = 0.006), sepsis and bacteremia (p<0.001), and pneumonia (p<0.001).

**Table 2:** Year-to-year changes in prevalence of vaccine and nonvaccine serotypes of *Streptococcus pneumoniae* in children after introduction of PCV7, Japan, April 2010–March 2013*

Disease and serotype	Isolates, no. (%)			p value
	2010, n = 300†	2011, n = 146‡	2012, n = 156§	
Meningitis				0.006
VT	42 (14.0)	17 (11.6)	6 (3.8)	
NVT	20 (6.7)	17 (11.6)	15 (9.6)	
Sepsis and bacteremia				<0.001
VT	111 (37.0)	42 (28.8)	13 (8.3)	
NVT	48 (16.0)	39 (26.7)	94 (60.3)	
Pneumonia				<0.001
VT	54 (18.0)	14 (9.6)	3 (1.9)	
NVT	8 (2.7)	6 (4.1)	21 (13.5)	
Other				0.147
VT	13 (4.3)	7 (4.8)	1 (0.6)	
NVT	4 (1.3)	4 (2.7)	3 (1.9)	
Total				<0.001
VT	220 (73.3)	80 (54.8)	23 (14.7)	
NVT	80 (26.7)	66 (45.2)	133 (85.3)	

*PCV7, 7-valent pneumoccal conjugate vaccine; VT, vaccine serotype (serotypes 4, 6B, 9V, 14, 18C, 19F, 23F); NVT, serotypes not included  in PCV7. †Voluntary vaccination with PCV7. ‡Implementation of Urgent Promotion of Vaccination incentive. §Just before the introduction of 13-valent PCV.

### Correlation between Serotype and Genotype

**Figure 2:**
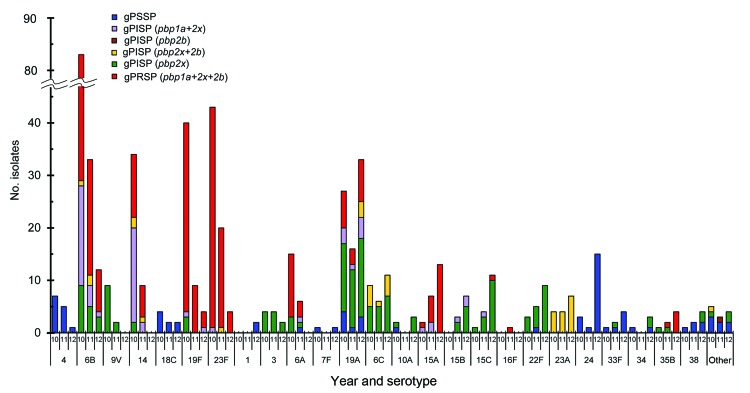
Changes in serotype number and penicillin resistance according to genotype, Japan, April 2010–March 2013. gPSSP, genotypic penicillin-susceptible *Streptococcus pneumoniae*; gPISP, genotypic penicillin-intermediate resistant *S. pneumoniae;* gPRSP, penicillin-resistant *S. pneumoniae*. The parentheses express abnormal *pbp* gene mediating penicillin resistance. 2010 indicates first surveillance period (April 2010–March 2011); 2011 indicates second surveillance period (April 2011–March 2012); 2012 indicates third surveillance period (April 2012–March 2013).

We also determined correlations between changes of serotypes and resistance genotypes in all 602 isolates for each period (Figure 2). These resistance genotypes were classified according to real-time PCR results concerning 3 PBP genes: *pbp1a*, *pbp2x*, and *pbp2b*.

Overall, gPRSP and gPISP (*pbp1a+pbp2x*), respectively, declined from 54.7% and 14.3% in 2010, to 47.3% and 8.2% in 2011, to 26.3% and 5.1% in 2012. Among them, vaccine serotypes 6B, 14, 19F, and 23F, including gPRSP and gPISP (*pbp1a+pbp2x*), which showed high frequency in 2010, decreased significantly during the 2 subsequent periods (p<0.001). Of serotypes contained in PCV13, serotype 6A, showing cross-protective immunity with 6B, also decreased markedly in 2012, but prevalence of serotype 19A increased. Serotypes 1, 3, and 7F included a very small number of isolates.

Coverage rate of PCV13 decreased rapidly from 89.0% in 2010 to 72.6% in 2011 to 39.1% in 2012 (p<0.001). Nonvaccine serotypes increased, especially serotypes 24 and 33F, identified as gPSSP; 15B, 15C; and 22F, gPISP (*pbp2x*); and 15A and 35B, gPRSP.

Main clonalities within major nonvaccine serotypes were identified as sequence type (ST) 3111 and ST2331 in serotype 19A, ST63 in serotype 15A, ST199 in serotypes 15B and 15C, ST433 in serotype 22F, ST338 of clonal complex (CC) 156 in serotype 23A, ST5496 of CC2572 in serotype 24, ST717 in serotype 33F, and ST558 in serotype 35B. STs in gPRSP among nonvaccine serotypes were as follows: major strains of serotype 15A belonged to ST63; serotype 15C belonged to ST83 of CC81; serotype 35B belonged to ST558; and serotype 16F belonged to ST8351 of CC3117.

### Yearly Changes in Macrolide Resistance

**Figure 3:**
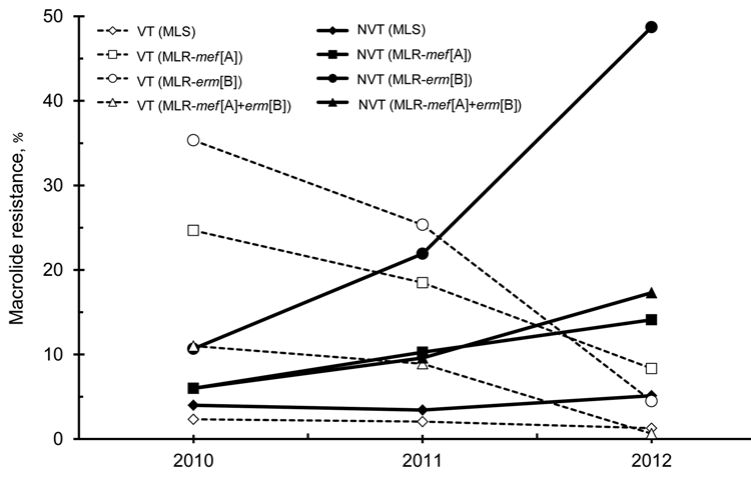
Proportional yearly changes in macrolide resistance according to resistance genes *erm*(B) and *mef*(A) identified by real-time PCR, Japan, April 2010–March 2013. The percentage of each resistance gene was calculated from the number of *Streptococcus pneumoniae* strains for each year. VT, vaccine serotype (serotypes 4, 9V, 18C, 6B, 14, 19F, 23F) included in the 7-valent pneumococcal vaccine; NVT, serotypes not included in the 7-valent pneumococcal conjugate vaccine; MLS, macrolide-susceptible strains not possessing any resistance gene; MLR-*mef*(A), macrolide-resistant strain possessing the *mef*(A) gene; MLR-*erm*(B), macrolide-resistant strain possessing the *erm*(B) gene; MLR-*mef*(A)+*erm*(B), macrolide-resistant strain possessing both *mef*(A) and *erm*(B) genes.

Macrolide resistance was classified into 4 groups: MLS, MLR-*mef*(A), MLR-*erm*(B), and MLR-*mef*(A)+*erm*(B). Comparing results between 2010 and 2012, proportions of resistance types of MLR-*mef*(A), MLR-*erm*(B), and MLR-*mef*(A)+*erm*(B) in vaccine serotypes decreased, whereas that of MLR-*erm*(B) in nonvaccine serotypes increased significantly (p<0.001) (Figure 3).

**Figure 4:**
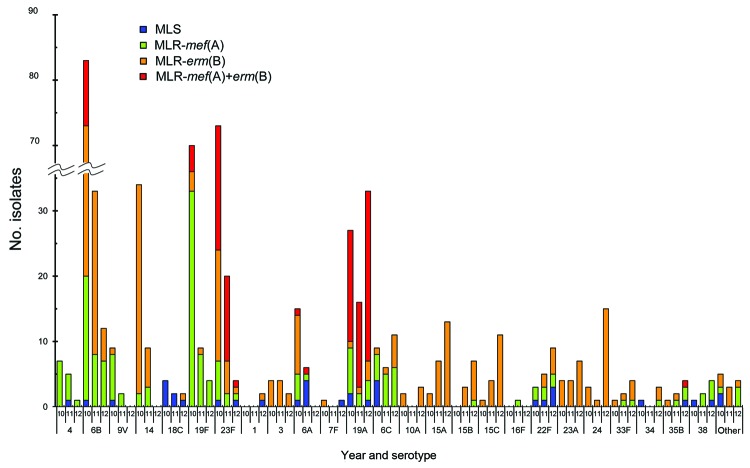
Changes in serotype number and macrolide resistance of *Streptococcus pneumoniae* strains according to genotype, Japan, April 2010–March 2013. MLS, macrolide-susceptible strains not possessing any resistance gene; MLR-*mef*(A), macrolide-resistant strain possessing the *mef*(A) gene; MLR-*erm*(B), macrolide-resistant strain possessing the *erm*(B) gene; MLR-*mef*(A)+*erm*(B), macrolide-resistant strain possessing both *mef*(A) and *erm*(B) genes.

Relationships between serotypes and macrolide resistance genes changed from year to year (Figure 4). In contrast to decreases in vaccine serotype strains possessing *erm*(B) or *mef*(A) genes, nonvaccine serotypes 15A, 15B, 15C, and 24, which possess the *erm*(B) gene mediating high macrolide resistance, increased. Almost all strains of serotype 19A possessed both genes *erm*(B) and *mef*(A).

### Changes of Relative Ratios of Every Serotype

As for increases and decreases in the proportion of each serotype isolated from patients <5 years of age from 2010 (vol-PCV7) to 2012 (pre-PCV13), vaccine serotype strains 6B, 14, 19F, and 23F, and 6A (which shows cross-protective immunity with 6B, occurring frequently among IPD cases) decreased markedly (p<0.001 for 4 vaccine serotypes, p = 0.005 for 6A). Serotype 19A, included in PCV13, increased (p<0.001). Other serotypes included in PCV7 or PCV13, except for 6A and 19A, changed minimally. Overall, proportions of nonvaccine serotypes not covered by PCV13 increased, particularly serotypes 15A, 15B, 15C, and 24 (each p<0.001), and 22F (p = 0.004).

### Patient Age and Vaccination History in 2012

Only 23 patients had infections caused by strains of vaccine serotypes (14.7%), 21 of whom had not received PCV7 (Table 3). Of the remaining 2 patients, a 1-year-old child infected with a strain of serotype 6B had received 2 doses of vaccine. The other patient, 5 years of age, was infected with a strain of serotype 19F; vaccination history was unknown.

**Table 3:** Pneumococcal vaccination history and age for 156 children with invasive pneumococcal disease, Japan, 2012*

No. vaccine doses administered	<6 mo	7–11 mo	1 y	2 y	3 y	4 y	5–9 y	>10 y	Total no. (%)
0		7 [3]	15 [8]	4 [1]	5 [2]	8 [3]	4 [1]	5 [3]	47 (30.1)
1			1		5	3	1		10 (6.4)
2	3		4 [1]	1	2	1			11 (7.1)
3	2	13	24	6					45 (28.8)
Booster			16	5					21 (13.5)
Unknown	1	2	11	3	2		1 [1]	1	22 (14.1)
Total	6	22	71	19	14	12	6	6	156 (100.0)
*Numbers in brackets indicate infections with a vaccine serotype.									

## Discussion

Introduction of PCV7 to prevent pneumococcal infections in children was credited with dramatic declines of the incidence of IPD in the United States (*6*,*7*), European Union countries (*24*,*25*), and many other nations (*26*).

During this implementation, increases in pneumococcal infections caused by serotype 19A and other nonvaccine serotypes, including those comprising many penicillin-nonsusceptible strains, has raised problems in clinical practice (*10*,*11*,*27*). Large-scale longitudinal surveillance showed that the rate of coverage by PCV7 decreased from 70% during 1999–2000 to 4.3% during 2008–2009 (*16*). In light of these observations, PCV7 was replaced with PCV13 in the United States in 2010 (*15*). After the change to PCV13, penicillin-nonsusceptible strains and serotype 15A or 35B strains increased as they did after PCV7 was introduced (*16*).

In Japan, PCV7 vaccination of children <5 years of age began at the end of 2010 under the Provisional Special Fund for the Urgent Promotion of Vaccination. This measure led to routine vaccination with PCV7 beginning in April 2013 until the vaccine was changed to PCV13 in November 2013. Nationwide, the estimated rate of PCV7 vaccination for children <5 years of age in 2012 and 2013 was 80%–90% and >90%, respectively. We examined the influence of PCV7 against IPD infection in children in detail with active cooperation from 341 clinical laboratories at general hospitals. We therefore believe that our data are highly likely to reflect changes in trends of serotypes and strains causing IPD after PCV7 introduction in Japan. Unfortunately, we could not calculate a precise incidence of IPD per 100 000 children.

The decrease of IPD cases caused by strains of vaccine serotypes after promotion of PCV7 contributed to a reduction in overall IPD by half. Furthermore, dramatic reductions in number of cases caused by serotypes 6B, 14, 19F, and 23F—including mainly gPISP and gPRSP—have been beneficial.

However, the relative proportion of IPD caused by nonvaccine serotypes, such as 15A, 15B, 15C, 19A, 22F, 24, and 35B, increased year by year, and the PCV7 coverage rate fell drastically from 73.3% in 2010 to 14.7% in 2012. The impact of routine PCV13 vaccination implemented in December 2013 is likely to be much less than that from PCV7 because coverage already had declined to 39.1% in 2012. However, infections caused by serotype 3, included in PCV13, are expected to decrease. Although IPD caused by serotype 3 occurs infrequently among children in Japan, serotype 3 remains important as a pathogen causing acute otitis media in children and pneumonia or empyema in elderly persons.

Two findings in our study stand out. First, gPRSP strains were confirmed in serotypes 15A, 15C, 16F, and 35B. In particular, strains of serotypes 15A and 35B in 2012 all represented gPRSP. According to multilocus sequence typing analysis, serotype 15A in this study belonged to ST63 of CC63, registered as Sweden 15A-25 in the Pneumococcal Molecular Epidemiology Network clone. Serotype 35B also belonged to ST558 of CC558 from the United States. In addition, gPRSP of serotype 15C corresponded to ST83 of CC81, previously reported from Taiwan. Only 1 serotype 16F strain representing gPRSP was newly identified as ST8351 of CC3117 in the present study. Increasing migration associated with economic development is spreading pneumococcal strains worldwide.

Our finding that vaccine serotype strains are being rapidly replaced by strains of nonvaccine serotypes, such as serotypes 15C and 24, was associated with increased high macrolide resistance mediated by the *erm*(B) gene. In this context, wide use of 14-membered and azalide macrolides for children, in addition to adults, will be a major factor favoring resistance. Excessive use of macrolides in Japan also has resulted in substantial increases of macrolide-resistant *Mycoplasma pneumoniae* (*28*) and macrolide-resistant *S. pyogenes* strains (*29*). This alarming problem suggests a need to strictly control macrolide use, beginning as soon as possible.

We could not analyze relationships between capsular type and death in children because deaths among children were extremely low (<2.2% every year). The low death rate reflects equal and easy access to hospitals because of Japan’s universal health insurance system.

Recently, recombination of the *cps* gene encoding capsular polysaccharides among pneumococcal strains with different capsular types, called capsular switching, was observed by Brueggemann et al. (*30*). Capsular switching also is associated with recombination of PBP genes, considering that *pbp1a* and *pbp2x* genes which mediate β-lactam resistance, are positioned at both ends of the *cps* region. Dissemination of vaccine and excessive use of antimicrobial agents could favor *S. pneumoniae* with a new capsular type in the future.

In conclusion, sustained surveillance on a national and international scale is needed to control pneumococcal infections, especially considering the multifaceted consequences of vaccination programs. Also, controlling the use of antimicrobial agents is urgently needed to avoid increases in the resistant pathogens.
